# Increased Porphyrins in Primary Liver Cancer Mainly Reflect a Parallel Liver Disease

**DOI:** 10.1155/2009/402394

**Published:** 2009-10-18

**Authors:** Jerzy Kaczynski, Göran Hansson, Sven Wallerstedt

**Affiliations:** ^1^Department of Medicine, Göteborg University, Sahlgren's University Hospital, SE-41685 Göteborg, Sweden; ^2^Department of Pathology, Göteborg University, Sahlgren's University Hospital, SE-41345 Göteborg, Sweden

## Abstract

Hepatic porphyries have been associated with an increased risk of primary liver cancer (PLC), which on the other hand may cause an increased porphyrin production. To evaluate the role of an underlying liver disorder we analyzed porphyrins in patients with hepatocellular carcinoma (HCC) (*n* = 65), cholangiocellular carcinoma (*n* = 3), or suspected PLC, which turned out to be metastases (*n* = 18) or a benign disorder (*n* = 11). None of the patients had a family history of porphyry or clinical signs of porphyry. Increased aminolevulinic acid or porphyrin values were common not only in patients with PLC (43%) but also in metastatic (50%) and benign (64%) liver disorders. The corresponding proportion for HCC patients with liver cirrhosis (55%) was higher (*P* < .05) than in those without cirrhosis (17%). We conclude that symptomatic porphyries are unusual in PLC, whereas elevated urinary and/or faecal porphyrins are common, primarily reflecting a parallel liver disease and not the PLC.

## 1. Introduction

Primary liver cancer (PLC), and particularly its most predominant histological type, hepatocellular carcinoma (HCC), is one of the most common malignancies in the world [1]. The incidence rates vary widely throughout the world [2]. Sweden is a low-rate area where PLC accounts for less than 2% of all diagnosed cancers [3]. Cirrhosis of the liver plays an important role for the PLC development, and important etiological factors for HCC are reported to be hepatitis B and C viruses, alcohol, and diabetes [2, 4–8]. 

Among other causes of PLC an association with hepatic porphyries has been recognized, especially with acute intermittent porphyry (AIP) [9–16] and porphyria cutanea tarda (PCT) [15, 17–22], but also with porphyria variegata [14, 16, 23] and hereditary coproporphyria [14, 16, 24]. A relation between AIP and HCC was first noted in northern Sweden [9] and was supported in additional studies from Scandinavia [10–13, 15, 25] and other countries [14, 16, 26, 27]. The incidence of HCC in PCT has been reported to be from zero% to 34% [20, 21, 28–30].

The most common type of PCT is an acquired form, which is triggered by toxic (e.g., alcohol) and infectious (e.g., hepatitis C virus) factors or haemosiderosis [28, 31, 32], and liver cirrhosis and hepatic iron overload are common associated findings [19]. Although each of these factors is known to increase the risk of HCC as well [5, 6, 33, 34], it has been suggested that PCT *per se* increases this risk further [18, 22]. 

Porphyry in patients with PLC as a paraneoplastic phenomenon is a well-documented but rare condition [19, 35, 36]. The reported cases had all photosensitive skin lesions, but in several instances the pattern of porphyrin excretion was not consistent with true PCT. Secondary AIP has also been reported [37]. Abnormalities in porphyrin metabolism without clinical picture resembling hepatic porphyries are also described in patients with liver tumours and other liver diseases [38, 39]. However, little is known about the frequency and excretion patterns of urinary and particularly faecal porphyrins in patients with PLC.

The aim of the present study was to determine the excretion of urinary and faecal porphyrins and their precursors by conventional methods in PLC patients in order to (i) reveal undiagnosed porphyry cases in order to elucidate the significance of porphyries for PLC, and (ii) examine whether elevated urinary and/or faecal porphyrins are associated with PLC *per se*, a parallel or another liver disease.

## 2. Material and Methods

### 2.1. Patients

This study was performed as a part of a prospective study of patients with PLC in Göteborg, Sweden. All patients who were admitted to hospital because of a strong suspicion of PLC and who came to the authors' attention were included in the study, which was ethically conducted in accordance with the principles of the 1983 Declaration of Helsinki. Since the diagnostic procedures did not made it possible to confirm or exclude PLC in five of the 109 patients primarily included in the study, they had to be excluded. In further 7 cases it was not possible to determine the excretion of porphyrins and their precursors, either because the sampling was not performed for various reasons, such as unexpected death, or if it was performed incorrectly or insufficient, and so forth. Of the remaining 97 cases (Table 1) the final diagnosis was PLC in 68 cases, malignancy other than PLC in 18 cases, and benign liver disorders in 11 cases. These latter 29 patients constituted control groups I and II in this study. 

All patients were interviewed (JK or SW) with regard to clinical or family history of porphyries, haemochromatosis, and chronic alcoholism. The patients were followed up by personal examination and by study of their clinical records.

### 2.2. Sampling and Biochemical Analyses

Within one week from the interview two samples of 24-hour urine and one sample of faeces were collected from each studied subject. One 10 mL sample of 24-hour urine was alkalized with 5 g sodium bicarbonate and analyzed with regard to the presence of porphobilinogen (PBG) with a qualitative method. The presence of elevated concentrations of porphyrins were analyzed in the same urine sample with a qualitative and/or quantitative method (spectrophotometry) and expressed as 0-1 (absence-presence), in *μ*mol/24 hour and/or mmol/mol creatinine. Aminolevulinic acid (ALA) was analyzed in another 24-hour urine sample after acidification with 30 mL hydrochloric acid, 5 mol/L. It was determined by ion-exchange chromatography and expressed in *μ*mol/24 hour and/or mmol/mol creatinine. Porphyrins in faeces were determined in a sample of about 5 g, by thin-layer chromatography. Coproporphyrins (CPs) and protoporphyrins (PPs) were expressed in nmol/g faeces. Transferrin saturation in serum was also measured in every patient.

### 2.3. Histopathological Examinations

The specimens for histopathological examination were obtained by biopsy ante mortem (needle, wedge biopsy or tissue blocks of resected tumours) or at autopsy. The formalin-fixed, paraffin-embedded tissue specimens were collected, and slides for light microscopy were stained with haematoxylin and eosin. To improve accuracy of the diagnosis, some cases were also stained with Grimelius stain (silver) and chromogranine and some were studied immunohistochemically by use of the peroxidase/anti-peroxidase (PAP) method for presence of alpha-fetoprotein, thyroglobulin, factor VIII, and prostate specific antigen. The slides of all cases with final diagnosis of HCC were also stained with Perl's Prussian blue stain for iron. Siderosis was graded as follows: (0) absent (1) minimal, (2) moderate, (3) abundant, and (4) massive. All the microscopic slides were reviewed by two of us (GH, JK) and studied also with regard to the diagnosis and the histology of available, nonneoplastic liver tissue.

PLC was classified according to Anthony and The international working party [40, 41]. In eight cases without available or insufficient tissue specimens, a diagnosis of HCC was established since they fulfilled at least two of the following three criteria: (1) diagnosis of HCC as said by cytological examination of a fine-needle aspiration specimen, (2) radiological suspicion of PLC, and (3) alpha-fetoprotein concentration in serum > 500 *μ*g/L [42].

### 2.4. Statistical Analyses

Standard statistical methods were employed, using the group comparison t test for comparison of two mean values and the chi-square or Fisher's exact test for comparison of two proportions.

## 3. Results

### 3.1. Patients

The mean age of the PLC patients at the time of inclusion was 68 years with no significant sex differences. The male to female ratio was 3 : 1 (Table 1). The patients with benign liver disorders were significantly younger than both PLC patients (*P* < .01) and patients in control group I (*P* < .05). Twenty-one of the 97 patients (22%) had a history of heavy alcohol consumption but there were no significant differences between the study groups. 

None of the patients had a clinical or family history of porphyry or haemochromatosis, nor did any of them exhibit any porphyry symptoms during the followup. All patients with PLC and metastatic liver disease died during the study period.

### 3.2. Histopathology

There were 65 cases of HCC and 3 cases of CCC. Cirrhosis of the liver could be established in 24 of 36 HCC cases (67%) in which nonneoplastic liver tissue was available for microscopic examination. In seven additional cases there was a strong clinical suspicion and/or typical macroscopic appearance of cirrhosis at autopsy. In 22 HCC cases, however, it was not possible to confirm or exclude cirrhosis clinically or histopathologically due to insufficient nonneoplastic liver tissue or autolytic changes. There was no case of massive siderosis in the nonneoplastic liver tissue and only four cases of moderate/abundant siderosis, all of which had cirrhosis and HCC. Alcohol and hepatitis C were the predominant causes of liver cirrhosis (Table 2). The cirrhosis was unknown before HCC diagnosis in 18 cases and well compensated in the remaining 13 patients. 

In the control group I, that is, 18 patients with a metastatic liver disease, there was one case with liver cirrhosis.

In the control group II, that is, 11 patients with benign liver diseases, there were four cases with liver cirrhosis, three with histological normal liver or steatosis, two with liver abscess, and one of each with peliosis hepatis and a benign liver tumour (fibrous histiocytoma).

### 3.3. Biochemistry

Totally, concentration values above the laboratory reference range of at least one of ALA, urinary porphyrins, CP, or PP in faeces, were found in 43% in the PLC group, 50% in group I, and 64% in group II. The differences between these groups were not significant. The corresponding figure for patients with HCC and liver cirrhosis (55%) was higher (*P* < .05) than for noncirrhotic patients with HCC (17%).

**ALA**

There was no significant difference in frequency of increased concentration of ALA in urine between the groups (Table 1). Most of these patients had only slightly increased concentrations (reference limit < 40 *μ*mol/24 h and < 3 mmol/mol creatinine). The highest values (120 *μ*mol/24 h and 26 mmol/mol creatinine) were found in a patient with HCC without cirrhosis. This patient had a concentration of porphyrins in urine and faeces within the reference range.

**PBG**

There was not a single case with qualitatively detectable urinary levels of PBG.

**Urinary Porphyrins**

The presence of increased concentrations of urinary porphyrins, measured qualitatively and/or quantitatively, was noted more often in HCC patients with than without liver cirrhosis (*P* < .05) (Table 3). The highest concentration of porphyrins, 1.4 *μ*mol/24 hour (reference limit < 0.6) and 0.26 mol/mol creatinine (reference limit < 0.04), was found in a patient with HCC and liver cirrhosis related to alpha-1-antitrypsin deficiency.

**Porphyrins in Faeces**

The finding of increased concentrations of CP and/or PP in faeces was common in both PLC patients (39%) and the controls (45% and 44%, resp.). The differences were not significant. In HCC patients it was more common (*P* < .05) with increased concentrations of CP and/or PP in faeces in those with underlying cirrhosis (59%) than in noncirrhotic HCC patients (11%). The highest value of CP, 64 nmol/g faeces (reference limit < 20 nmol/g), was found in a 71-year-old patient with modest alcohol consumption and a high serum concentration of alpha-fetoprotein (>10000 *μ*g/L). Due to autolytic changes it was not possible to state a histopathological diagnosis. At autopsy, however, the liver had a typical macroscopic appearance of cirrhosis and HCC. The highest values of PP, 750 and 760 nmol/g faeces (reference limit < 70 nmol/g), were found in a patient with alcoholic liver cirrhosis without HCC and in a patient with liver cirrhosis of unknown aetiology with HCC, respectively. The latter patient had also the highest values of CP but values of ALA and porphyrins in urine within the reference range.

There was no significant difference in frequency of increased urinary and/or faecal porphyrins between alcoholics (43%) and other patients and (47%).

**Siderosis of the Liver and Transferrin Saturation**

One of the four patients HCC with liver cirrhosis and siderosis had increased concentrations of ALA and urinary porphyrins. Two of these four patients and additional two had high (>55%) transferrin saturation. They were all alcoholics. One had increased concentrations of ALA and urinary porphyrins (same patient as above) and one had increased concentrations of urinary porphyrins and PP in faeces.

## 4. Discussion

Our finding of a substantial number of PLC patients with increased concentrations of urinary and/or faecal porphyrins should not be surprising, since porphyries are not unusual in liver disease [28, 31, 32, 38, 39]. The characteristics of the PLC patients in our study with regard to frequency of cirrhosis, age, and sex are in agreement with findings in a large comprehensive retrospective study from the same area and should thus be regarded as representative for PLC in Sweden [43]. It thus seemed appropriate to compare porphyrin divergences in our PLC cases not with healthy people but with patients suffering from other liver disorders, especially patients with clinical signs suspect of PLC. Since the frequency of elevated porphyrins, to our knowledge, has not been studied in metastatic liver disease, the control group was divided in patients with either a benign or a malignant (other than PLC) liver disorder. 

There was not a single case of AIP or PCT in our study, which indicates that porphyries generally play a minor etiologic role for PLC. The finding of increased PBG in urine in patients with PLC has been reported [44] but seems to be rare. This is in accordance with the results in our study. However, we cannot rule out the possible etiological role of AIP in the development of PLC in single cases, since the prevalence of this disease is low and only about one third of the AIP gene carriers have increased PBG levels in the urine [45]. 

An acquired form of PCT has been associated with various liver disorders such as cirrhosis, and hepatitis C virus infection [28, 38, 46]. The majority of PLC cases arise in cirrhotic livers [2] and hepatitis C virus is one major etiologic factor for HCC [5, 40]. Thus, most of the cases of PCT with PLC seem to be the result of the same liver disorder. 

None of the patients in our study developed a clinical picture resembling hepatic porphyry. The patients with elevated porphyrins had a pattern of ALA and porphyrin excretion in urine and faeces, different from that observed in AIP and PCT. Since the prognosis for PLC is usually very poor [47–49], the time would be too short for accumulation of porphyrins to levels, giving clinical porphyry symptoms [38]. In our study the median survival rate from the time of the diagnosis was two months.

The finding of elevated urinary and/or faecal porphyrins, without exhibiting clinical symptoms resembling porphyries, was common in PLC patients in our study. However, this finding was also common in both patients with metastatic and benign liver diseases. Since there was no characteristic pattern of ALA and porphyrins excretion in PLC, increased porphyrin secretion may indicate a liver disorder but not specifically PLC.

Our results suggest that elevated urinary and faecal porphyrins are common in many liver disorders, irrespective of if they are malignant or not. Since the liver plays a dominant role in haeme synthesis [50], it is not surprising that functional morphological disturbances and malignant degeneration may lead to disorderly haeme synthesis, not unlike the porphyries [18, 38, 51]. The possible subsequent clinical manifestation seems to depend on level and constellation of porphyrin accumulation and excretion [38]. Thus, in one study almost one-third of all patients with chronic liver damage showed a pathological porphyrinuria [38]. However, porphyrinuria is often transient and reversible [39].

Whatever the mechanism, both PLC and other liver disorders may lead to increased ALA and/or porphyrins synthesis, as shown in our study. This chronic porphyry presents a variable constellation of ALA and porphyrin excretion pattern in urine and faeces.

We conclude that although increased ALA and porphyrin values in urine and/or faeces in PLC patients were found to be frequent, this seems to cause symptomatic porphyries only in exceptional cases. Since increased porphyrin values were common especially in HCC patients with concomitant cirrhosis, and also in other (malignant and benign) liver diseases, these disturbances probably primarily reflect an underlying, parallel or another liver disease and are not characteristic for PLC *per se*.

## Figures and Tables

**Table 1:** Proportion of evaluable patients (%) with elevated urinary and/or faecal porphyrins measured as concentration of a metabolite above the laboratory reference range (Upper limit of normal, ULN) in cases with primary liver cancer, metastatic liver disease, and benign liver disorders. ALA : aminolevulinic acid; PBG : porphobilinogen.

	Primary liver cancer	Metastatic liver disease	Benign liver disorders
	*n* = 68	*n* = 18	*n* = 11
Age (years, range)	68 (49–92)	67 (39–81)	54 (27–74)
Sex (M/F)	51/17	9/9	4/7
U-ALA (% above ULN)	11^3^	29^5^	27
U-PBG (% above ULN)	0^2^	0^5^	0^8^
U-Porhyrins (% above ULN)	15^1^	12^5^	13^9^
F-Coproporhyrins (% above ULN)	20^4^	30^7^	50^9^
F-Protoporphyrins (% above ULN)	35^4^	45^6^	33^8^

^1^ *n* = 65, ^2^ *n* = 64, ^3^ *n* = 61, ^4^ *n* = 49, ^5^ *n* = 17, ^6^ *n* = 11, ^7^ *n* = 10, ^8^ *n* = 9, ^9^ *n* = 8.

**Table 2:** Etiology of cirrhosis in 31 patients with HCC.

Etiology	*n*	%
alcohol^1^	13	42
hepatitis C^1^	7	23
autoimmune hepatitis	1	3
primary biliary cirrhosis	1	3
alpha-1-antitrypsin deficiency	1	3
NASH	1	3
hepatitis B	0	0
unknown	9	29

^1^includes 2 cases with history of both alcohol abuse and hepatitis C.

**Table 3:** Frequency of elevated urinary and/or faecal porphyrins in 43 patients with hepatocellular cancer (HCC), where presence of cirrhosis could be established. In each column is given the proportion (%) of evaluable patients with concentrations above the laboratory reference limit (Upper limit of normal, ULN). ALA : aminolevulinic acid; PBG : porphobilinogen; NS : non significant.

	HCC with cirrhosis	HCC without cirrhosis	Difference between groups
	*n* = 31	*n* = 12	
Age (years, range)	68 (49–83)	65 (51–72)	
Sex (M/F)	27/4	6/6	
U-ALA (% above ULN)	7^1^	8^4^	NS
U-PBG (% above ULN)	0^2^	0^4^	NS
U-Porhyrins (% above ULN)	28^1^	0^4^	*P* < .05
F-Coproporhyrins (% above ULN)	36^3^	9^5^	NS
F-Protoporphyrins (% above ULN)	55^3^	9^5^	*P* < .05

^1^ *n* = 29, ^2^ *n* = 28, ^3^ *n* = 22, ^4^ *n* = 12, ^5^ *n* = 11.
